# Lasso Peptides—A New Weapon Against Superbugs

**DOI:** 10.3390/ijms26178184

**Published:** 2025-08-23

**Authors:** Piotr Mucha, Jarosław Ruczyński, Katarzyna Prochera, Piotr Rekowski

**Affiliations:** Laboratory of Biologically Active Compounds Chemistry, Department of Molecular Biochemistry, Faculty of Chemistry, University of Gdansk, Wita Stwosza 63, 80-308 Gdansk, Poland; jaroslaw.ruczynski@ug.edu.pl (J.R.); katarzyna.prochera@phdstud.ug.edu.pl (K.P.); piotr.rekowski@ug.edu.pl (P.R.)

**Keywords:** lasso peptides, multi-drug-resistant bacteria, post-translationally modified peptides, peptide synthesis, macrolactam ring, superbugs, methicillin and vancomycin-resistant strains

## Abstract

The emergence of multi-drug-resistant bacteria (known as superbugs) represents one of the greatest challenges for human health and modern medicine. Due to their remarkable ability to rapidly develop resistance to currently used antibiotics, new molecular targets for bacteria and substances capable of effectively combating related infections are still being sought. Lasso (known also as lariat) peptides are an unusual subclass of ribosomally synthesized and post-translationally modified peptides (RiPPs) with a structurally constrained knotted fold resembling a lasso. They are synthesized by certain groups of microorganisms as a result of complex processes involving intricate structural changes leading to the formation of the lasso structure. Reproducing these processes using known peptide synthesis methods poses a major challenge for synthetic chemistry. Lasso peptides exhibit a range of bioactivities including antibacterial activity. Due to the lasso structure, the peptides are capable of binding to new molecular targets, including atypical sides of ribosomes, in relation to currently used antibiotics. Thus, creating new mechanisms that inhibit metabolic processes leading to the death of pathogenic bacteria. This feature makes lasso peptides a potential “last chance” weapon in the fight against emerging superbugs.

## 1. Introduction

The emergence of multi-drug-resistant (MDR) bacteria, also known as superbugs, represents one of the greatest challenges for modern medicine. The estimated data analysis forecasts that superbugs are projected to cause millions of deaths globally, with estimates reaching 39 million by 2050 [1]. Antibiotics are essential drugs used for bacterial infection treatment, but the growing problem of bacterial resistance has rendered many pharmacological treatments ineffective [2,3]. The process of ever-increasing numbers of multi-drug-resistant bacterial strains is being monitored with concern by the WHO. The 2024 WHO Bacterial Priority Pathogens List (WHO BPPL) is an important tool in the global fight against multi-drug antimicrobial resistance [4]. As a result, 24 pathogens including Gram-negative bacteria resistant to last-resort antibiotics, drug-resistant *Mycobacterium tuberculosis*, and other high-burden resistant pathogens such as *Salmonella*, *Shigella*, *Neisseria gonorrhoeae*, *Pseudomonas aeruginosa*, and *Staphylococcus aureus* are on this list. Unfortunately, on each newly published WHO BPPL list, this number gets higher and higher. Effective defense mechanisms enable them to successfully defend themselves against known antimicrobial drugs, including so-called “antibiotics of last resort”. For this reason, new chemical compounds, metabolic pathways or molecular targets are being sought for use against superbugs. One promising group of compounds with strong antibacterial properties are lasso peptides. Lasso peptides are a unique subclass of RiPPs with a structurally constrained knotted fold resembling a lasso [5,6,7,8,9].

Generally, peptides are relatively small molecules among other biopolymers, consisting of a few to several dozen amino acid residues (AA) [10]. They arise as a result of the products of proteolytic hydrolysis of proteins synthesized via translation on ribosomes [11]. Non-ribosomal peptide synthesis (NRPS) pathways are also known. However, this mechanism is much rarer and characteristic of some lower organisms, such as bacteria [11]. Due to their short chain length, most peptides (without structural stiffeners) are characterized by high conformational freedom, which contributes to the fact that they usually do not have a defined and ordered spatial structure, adopting random coil conformation [12,13,14]. Some peptides contain one or more disulfide bridges in their structure, which are examples of post-translational modifications (PTMs) that stiffen the structure and limit the dynamics of the entire molecule [15,16,17,18,19,20]. There are also known, albeit relatively rare, examples of cyclic peptides (head to tail type) synthesized in lower organisms by non-ribosomal means [21]. Lasso peptides are completely different from “typical peptides” in terms of conformation/structure as well as physicochemical characteristics [5,6,7,8,9]. They are a unique class of ribosomally synthesized and post-translationally modified peptides (RiPPs) characterized by a C-terminal tail of the peptide chain trapped in a macrolactam ring forming a slip knot-like shape synthesized by certain groups of simple microorganisms (some strains of bacteria and archaea) [5,7].

The first peptide discovered and described in 1992 belonging to the lasso peptide group and currently considered the archetypal structure of this class of compounds was microcin J25 (MccJ25) [22]. Formally, anantin (currently classified as a lasso peptide) was identified a year earlier in 1991, but described “only” as a cyclic, not a lasso peptide [23]. While bioinformatics analyses predict thousands of lasso peptide sequences, roughly 50 distinct lasso peptides have been isolated, purified, and structurally characterized (Table 1).

Genome mining enabled the discovery of over 90 new lasso peptides [68,69]. These data suggest that the lasso peptides known so far are just the tip of the iceberg protruding from a sea of potential sequences hiding in bacterial genomes. Lasso peptides are formed as a result of an expression of a cluster of genes encoding both the lasso peptide sequence itself, auxiliary sequences, and sequences of enzymes involved in the post-translational processing of the precursor protein, which ultimately leads to its proteolysis and the formation of the lasso structure [5]. Recreating such synthesis using modern organic chemistry methods, especially commonly used peptide synthesis methods, is a major and, to date, virtually unsolved challenge. The presence of an atypical rigid structural element like a “lasso” is responsible for the unique properties of lasso peptides. The peptides possess remarkable thermal and proteolytic stability in the physiological environment, which make them good candidates for cargo molecules after conjugation with other bioactive molecules [5]. Also, a broad spectrum of biological activity such as antimicrobial activity, anticancer, and antiviral properties or the enzyme inhibition of lasso peptides is known [5,7]. Recent studies show that lasso peptides, by binding to the unique molecular targets of multi-drug-resistant bacteria (superbugs), extremely effectively block their main metabolic pathways, leading to cell death and thus inhibiting infection [5,7]. These make them attractive candidates for pharmaceutical research to make lasso peptides a potential “last chance” weapon against emerging superbugs. This review aimed to describe the current knowledge on the structural, synthetic, and antibacterial properties of lasso peptides.

## 2. Lasso Peptide Structure

**Figure 1:**
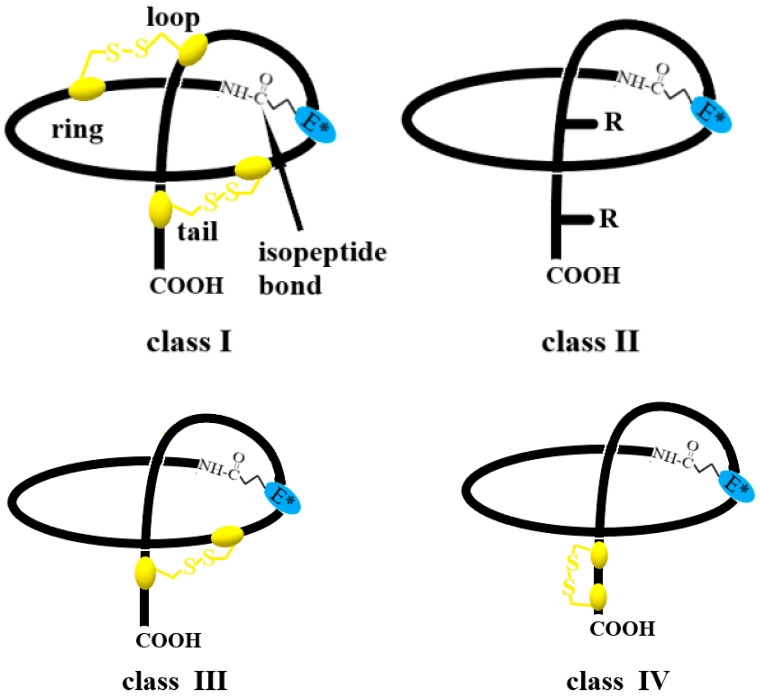
Schematic representation of structures of different classes (I–IV) of lasso peptides. The isopeptide bond formed by the N-terminal ε-amino group and the β-carboxyl group derived from the side chain of glutamic acid (E, blue oval) closing the macrolactam ring structure is shown. E*—in some lasso peptides, the E (Glu) residue may be replaced by an aspartic acid residue (D, Asp). Cysteine residues and disulfide bonds (-S-S-) are marked with a yellow color. R—amino acid residues with bulky side chains preventing the tail from slipping out of the macrolactam ring.

“Typical” lasso peptides are relatively short and compact peptides of 16–21 amino acid (AA) residues [5]. However, shorter (e.g., huascopeptin 13 AA, chaxapeptin, 15 AA) as well as longer (e.g., astexin-1, 23 AA; astexin-3, 24 AA; pandonodin 33-AA) lasso peptides are also known [7,8,70,71]. Their most characteristic structural element is a macrolactam ring, created by the formation of an isopeptide bond between the N-terminal amino group of the peptide chain and the β- or γ-carboxyl group of the side chain of aspartic acid (Asp, D) or glutamic acid (Glu, E) (Figure 1) [5]. During the ring closure, the *C*-terminal part of the peptide chain (tail) is closed and locked inside, creating a functional lasso structure (Figure 1).

Due to the stability and ability to permanently close the tail inside the ring, its optimal size is eight to nine amino acid residues [5,71]. In the case of an optimally sized macrolactam ring containing eight amino acid residues, when the β-carboxyl group Asp is involved in the ring closure, 22 atoms are locked in the ring (for γ-COOH Glu, 23 atoms). In the smaller macrolactam ring, it is extremely difficult to close the C-terminal tail for steric reasons. Although there are known examples of peptide lasso (e.g., huascopeptin) containing seven amino acid residue members of the ring [72]. Macrocycles with larger sizes (>9 AA) are too flexible and are not tight enough to permanently encapsulate the peptide’s lasso tail [5].

Disulfide bridges are an additional structural element, in addition to the macrocycle, that stabilize the peptide lasso [5,8]. The presence (or lack) of disulfide bridges is a structural element that allows lasso peptides to be divided into four classes (Figure 1). Lasso peptides containing two disulfide bonds are defined as class I. Those without any disulfide bridges are classified as class II. In this case the lasso structure is stabilized by the bulky side chains of amino acid residues within the C-terminal tail [5,7,8]. These residues act as steric locks called plugs and prevent the C-terminal tail from being unthreaded. In the case of Class III lasso peptides, the disulfide bridge connects the tail sequence with the macrolactam ring. Class IV contains a single disulfide bridge located in the C-terminal tail sequence [5,52]. This single disulfide bond located in the C-terminal sequence of the tail, on the one hand, increases the stability of the peptide and, in addition, acts as a steric hinge to prevent the tail from slipping inside the macrocycle. Recently, it has been proposed that lariocidin B (LAR-B), containing an additional isopeptide bond linking the macrolactam ring to the C-terminus of the tail, became the first representative of a new class (class V) of lasso peptides (Table 1) [49].

## 3. Biosynthesis of Lasso Peptides

**Figure 2:**
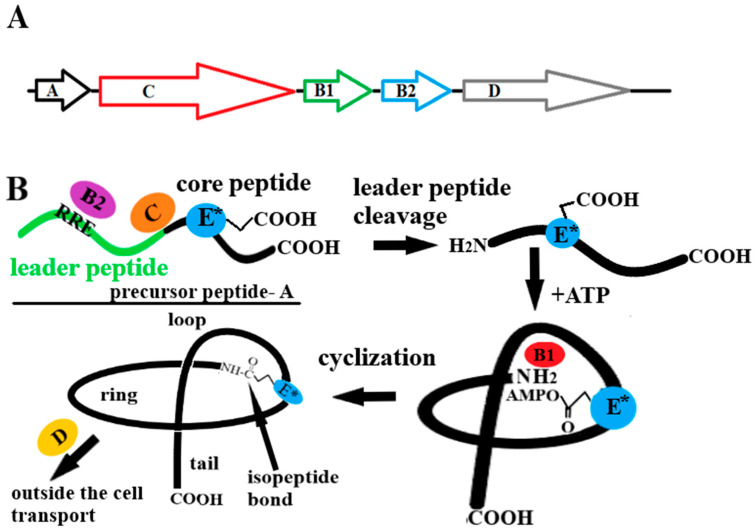
A typical gene cluster (encoding a separate RRE protein (B2) and leader peptidase (C)) involved in lasso peptide biosynthesis (**A**) and a scheme of lasso peptide biosynthesis (**B**). A—precursor lasso peptide, B1—lasso cyclase, B2—RRE protein, C—leader peptidase, D—ABC transporter, RRE—RiPP recognition element sequence, E*—glutamic acid (Glu) residue may be replaced with aspartic acid (D, Asp) residue.

Peptide lasso biosynthesis is a complex and multi-step process [5,7,73]. It occurs as a result of the expression of a cluster of genes encoding both the sequence of the lasso peptide and several enzymes responsible for its post-translational modifications and ultimately obtaining the lasso structure (Figure 2) [5].

The lasso peptide is originally synthesized as a larger precursor peptide which is composed of the N-terminal leader peptide and the C-terminal core peptide. The N-terminal leader peptide is responsible for the substrate recognition via the RiPP precursor recognition element (RRE) and the interaction with the PTM processing enzymes, while the core peptide is where the PTMs are introduced [71]. In the first step, the RRE protein recognizes and binds to the RRE motif of the N-terminal leader sequence of the precursor peptide, and then a leader peptidase (being a cysteine protease) cuts off the leader sequence. This releases the core sequence of the peptide, which undergoes further post-translational modifications. In the next step, lasso cyclase, using ATP, activates the β- or γ-carboxyl group of Asp or Glu, respectively [74]. The resulting active AMP-ester reacts with the N-terminal amino group of the core sequence, forming an isopeptide bond and closing the macrocycle structure [7,75]. Simultaneously, the C-terminal tail of the core sequence is threaded through the formed macrocycle and finally the lasso structure is formed. Prefolding leading to the proper orientation of the tail relative to the closing lactam ring is crucial to the formation of the lasso structure, since once the ring is closed, there is no way to thread the tail through the ring [76]. The lasso structure is additionally stabilized by the steric hindrance between the ring and bulky side chains of amino acid residues located within the C-terminal tail [77]. In the case of lasso peptides belonging to classes I, III, and IV, disulfide oxidoreductases form disulfide bridge(s) to further stabilize the lasso structure of the peptide. In the final step, the ABC transporter binds to the peptide lasso and exports it outside the bacterial cell. This suggests an extracellular function (e.g., antibacterial) of the peptide.

## 4. Approach to Chemical Synthesis of Lasso Peptides

Currently, there is no universal synthetic approach using known methods of organic synthesis, in particular solid-phase peptide synthesis (SPPS) for the synthesis of lasso peptides. The biosynthesis and availability of lasso peptides have been expanded through the development of heterologous expression and cell-free biosynthesis systems [75,78,79]. There are known examples of the synthesis of chemically modified lasso peptides using synthetic biology methods. An example of this approach is the synthesis of capistruin analogs, where supplementation-based incorporation and stop-codon suppression approaches were used for the co-translational incorporation of noncanonical amino acids into the lasso peptide, capistruin [80]. They enable the introduction of non-protein amino acids into the peptide chain, which are useful for studying the relationship between peptide structure and activity. They also enable the further modification of the chemical structure of the peptide using classical organic chemistry methods (e.g., click reaction). However, such methods are both time-consuming and costly. They require the use of synthesis methods that are difficult to automate, as well as final product purification, and their efficiency is low. The main limitation of a chemical synthesis of lasso peptides is the simultaneous closure of the macrolactam ring combined with the simultaneous closure of the C-terminal tail of the peptide inside it. While the cyclization of the ring itself through the formation of an isopeptide bond is a routine procedure in chemical peptide synthesis, doing so in such a way that the peptide tail is placed inside the ring is a huge synthetic challenge. In the biosynthesis of lasso peptides, this problem is solved by the appropriate pre-folding of the core peptide chain, enforced by interactions with PTM enzymes [5,7]. Relatively high hydrophobicity leading to problems with aggregation and solubility is another problem in the synthesis of lasso peptides [81]. While the commonly used method in peptide synthesis, SPPS, copes well with the low solubility of hydrophobic peptides thanks to the use of solvents such as N,N-dimethylformamide (DMF) or N-methylpyrrolidone (NMP), the aggregation process that occurs during synthesis poses many problems. High hydrophobicity is also a serious obstacle in peptide purification. Despite these synthetic difficulties, there are known examples of the use of known peptide synthesis methods in the synthesis of lasso peptides. Previous attempts to chemically synthesize lasso peptides have yielded meager results. Admittedly, it has been possible to synthesize a lasso peptide scaffold that was mimicked by the synthetic peptide-based rotaxane with a crown ether ring [82].

**Figure 3:**
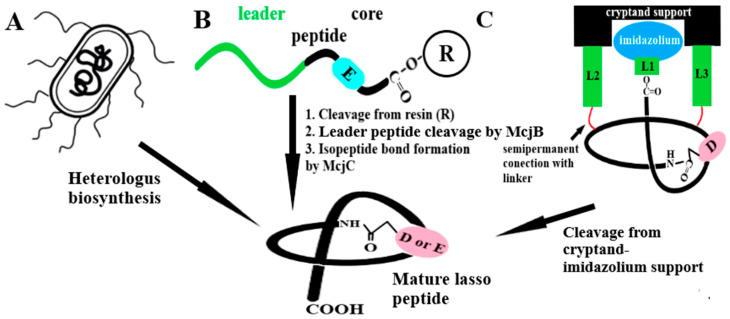
Lasso peptide synthesis strategies. (**A**)—heterologous (usually *E. coli* is used) biosynthesis [71], (**B**)—flow SPPS combined with enzymatic maturation [83], (**C**)—cryptand-imidazolium support SPPS [86].

An interesting proposal for the synthesis of lasso peptides was put forward by Hartrampf [83]. A hybrid chemoenzymatic method for the synthesis of MccJ25 (21 AA) consisting of fast flow SPPS combined with the enzymatic maturation of the precursor peptide McjA (57 AA) was developed. In the first stage, the McjA peptide, which is relatively long for chemical synthesis, containing the leader sequence (36 AA) and the core lasso peptide MccJ25 (21 AA), was synthesized chemically by SPS (Figure 3B). Then, after being cleaved from the resin and purified by chromatographic methods, the peptide was subjected to enzymatic maturation. Using the recombinant leader peptidase McjB, the leader sequence was cleaved, and then using a second recombinant enzyme, McjC lasso cyclase, an 8 AA macrolactam ring was formed by creating an isopeptide bond between the N-terminal α-amino group of G1 residue and the β-COOH of the side chain of E8 residue (Table 1), while threading the peptide tail sequence through the ring (Figure 3B).

The proposed strategy has undoubted advantages. The main one is the possibility of the virtually unlimited exploration of the activity–structure relationship (SAR) of lasso peptides, as the SPPS methodology allows for the inclusion of any protein and non-protein amino acid residue in the sequence, as well as modifications to the peptide chain itself [17,84,85]. An additional advantage is the possibility of synthesizing the precursor peptide in theoretically any quantity. On the other hand, however, the chemical synthesis of such long peptides (57 AA) is costly. The leader peptide (36 AA), although necessary for the maturation of MccJ25, serves only as an auxiliary element and, after being cut off, constitutes a specific and costly waste product in peptide lasso synthesis.

An equally costly stage is the maturation process of the peptide lasso. It requires the presence of two recombinant enzymes, McjB and McjC, for which the design of their expression system, biosynthesis, and finally purification of the obtained proteins are lengthy and costly stages [83]. Both enzymes are specific only to MccJ25 and cannot be used for other lasso peptides. This makes the maturation stage specific to MccJ25 and prevents it from being universal in the synthesis of any lasso peptide.

In 2019, an interesting new concept for the chemical synthesis of the peptide lasso was presented. Using a cryptand-imidazolium complex as a multi-semipermanent linker support, in which the imidazolium cation offers the first linker for the C-terminal tail formation of the lasso peptide and the cryptand furnishes the other two linkers for the N-terminal ring formation, the lasso peptide labeled as BI-32169 was synthesized [86] (Figure 3C). Their concept was based on starting the synthesis of the peptide lasso from the C-terminus, as in classical SPPS, however, using the imidazole cation as a carrier (first linker). All AAs used in the synthesis were, respectively, blocked in the side chains and linked together using the methods used in classical SPPS. After the first few AAs were attached, the imidazole support containing the residual C-terminal tail sequence was complexed with the cryptand. Subsequently, the entire C-terminal tail of the peptide and the initial fragment of the sequence included in the macrolactam ring containing the key D (Asp) residue forming the isopeptide bond were synthesized (Figure 3C). One of the initial AAs entering the ring was attached to the linker located on the cryptand (the second linker), and the attachment of subsequent AAs continued. Then one of the AAs included in the ring sequence was joined to the second linker of the cryptand (third linker) and the synthesis of the fragment included in the 9-AA ring was completed. The N-terminal end of the peptide was then linked to Glu via the isopeptide bond. This closed the macrolactam ring while maintaining the peptide’s binding to the three imidazole-cryptand support linkers. The N-terminal end of the peptide was then linked to Glu via an isopeptide bond. This closed the macrolactam ring while maintaining the peptide’s binding to the three imidazole-cryptand support linkers. The peptide was then dissociated from the two cryptand support linkers, and in the next step from the imidazole support linker. In the final step, a disulfide bridge was formed between the C-terminal residue of the Cys tail and that of the macrolactam ring. In this way, a peptide with a classical lasso structure containing a single disulfide bridge (class III) was obtained [86]. Using the strategy described above, the authors managed to synthesize both the *L*- and *D*-enantiomer of the lasso peptide BI-32169 and compare their properties. This shows the advantage of chemical methods that allow the easy incorporation of non-protein amino acids (including *D*-enantiomers) into the peptide chain, as opposed to the ribosomal synthesis pathway, which (except for special sophisticated techniques using stop codon) uses only protein amino acids (*L*-enantiomers). The strategy used by the authors is extremely ingenious and elegant, and most importantly, effective. However, it should be noted that the authors synthesized a lasso peptide containing a 9-AA macrolactam ring. This is the largest and least tight of the known stable lasso-type ring and at the same time the easiest to cyclize. The example of the chemical synthesis of the lasso peptide BI-32169 shows that chemical methods will in time supplant biological methods in the synthesis of lasso peptides and allow a far broader exploration of the relationship between their structure and activity than is currently the case. Figure 3 summarizes the strategies used in lasso peptide synthesis.

All methods designed to date are characterized by a lack of universality. Each of them is “tailored” to a specific lasso peptide. The heterologous biosynthesis of lasso peptides allows for the production of lasso peptides, but it is certainly not the target method for obtaining them. SPPS is a standard method used both for typical linear sequences and for peptides with complex topology. The bottleneck for this method is the stage of macrolactam ring cyclization with the simultaneous threading of the lasso peptide tail through it. The proposed strategy using cryptand-imidazolium support combined with the SPPS method, due to its complexity, can only be considered a first step towards finding a more universal method.

## 5. Antibacterial Activities of Lasso Peptides

The fact that lasso peptides are exported by ABC transporters outside the bacterial cell, coupled with data obtained from genome mining showing that lasso peptides are broadly distributed across the bacterial kingdom, suggested that the peptides were an important factor allowing interaction with the external environment [5,87,88]. Many reports in the literature show that lasso peptides do indeed have antimicrobial properties that make them a potential new weapon in the fight against bacterial infections, including those caused by drug-resistant strains [5,7,86]. The unusual features of lasso peptides make them promising candidates for a new generation of antibiotics. Their unique lasso structure combined with the tight structure allows the peptides to resist degradation in harsh physiological environments while ensuring extended antimicrobial activity [5]. The majority of lasso peptides exhibited antibacterial property against a broad range of bacterial strains. Archetypal MccJ25 is active against Gram-negative bacteria such as *Salmonella*, *Shigella*, *E. coli*, and *Enterobacter* species, while others like chaxapeptin and streptomonomicin target Gram-positive bacteria [5]. The peptides target different molecular targets by blocking key metabolic pathways responsible for bacterial cell function. MccJ25 and the newly discovered citrocin, inhibit the activity of bacterial RNA polymerase, thereby blocking transcription [89,90,91]. Some lasso peptides disrupt bacterial membrane integrity and permeability, contributing to their antibacterial properties. This is the case with MccJ25 what inhibits the activity of the membrane respiratory chain [89]. This is the second antibacterial mechanism of this peptide, alternative to RNAP inhibition. MccJ25, interacting with the bacterial membrane, disrupts the membrane respiratory chain, thereby inhibiting oxygen consumption and enabling the overproduction of superoxide, which ultimately leads to cell death.

In 2025, a new peptide from the lasso peptide group with broad-spectrum microbiological activity was discovered in *Paenibacillus* sp. M2. It was named lariocidin (LAR) [49]. Lariocidin is an 18-peptide with a characteristic lasso structure consisting of a macrolactam ring comprising an 8-AA ring and a 10-AA C-terminal fragment of the chain comprising a loop and a tail. Asp (D) is the key amino acid residue involved in the formation of the isopeptide bond. Although lariocidin’s structure is typical of lasso peptides, its mechanism of antimicrobial action is unique. LAR is the first lasso peptide that inhibits bacterial growth by binding to the ribosome and interfering with the translation process. Targeting the bacterial ribosome in a novel way that enables the bypassing of existing antibiotic resistance mechanisms. The peptide binds at a unique site in the small ribosomal subunit (30S), where it interacts simultaneously with the 16S rRNA and aminoacyl-tRNA. The interaction inhibits mRNA translocation and induces miscoding which consequently leads to the inhibition of bacterial protein biosynthesis. It seems that lariocidin relies on interactions with the RNA backbone rather than nucleobases. Such recognition of shape rather than sequence of the molecular target, which is 16S rRNA, may explain the fact that the peptide shows a strong tendency to evade common resistance mechanisms, including mutations that confer resistance to other drugs [49]. LAR displays a broad spectrum of antibacterial activity against both Gram-positive and Gram-negative bacteria, including those identified as multi-drug-resistant (MDR) or superbugs strains (Table 2) [49].

In in vitro experiments conducted at MHB in medium, LAR exhibited broad range activity against difficult and clinically challenging-to-treat strains with MICs in the tens (except for the *A. baumannii* ATCC17978 reference strain) µg/mL [49]. In nutrient-limited medium RPMI-1640, which better mimics in vivo conditions, it proved to be even more toxic to these bacterial strains. MIC values decreased by several dozen to several hundred times to values ranging from 0.5 to several µg/mL (Table 2) [49]. Importantly, under the conditions of the experiment, LAR showed no cytotoxicity against human cells or hemolytic activity [49]. Due to MDR and carbapenem-resistant properties, *A. baumannii* C0286 was used in in vivo antibacterial activity studies of LAR. Treatment of mice infected with this strain resulted in a significant (2 orders of magnitude) reduction in bacterial burden in the spleen, thigh and blood at 24 h after the administration of LAR [49]. All infected mice survived more than 48 h after LAR treatment, while none of the control group survived 28 h. The results of in vitro and in vivo experiments are a good predictor for the use of LAR, the first lasso peptide binding to the unique site on the bacterial ribosome, in the treatment of opportunistic bacterial infections.

Recently, it has been shown that other lasso peptides such as Sviceucin and Siamycin I also exhibit a strong bactericidal effect on superbugs like vancomycin (VA)-resistant enterococci (VRE) [92]. Sviceucin and Siamycin I used independently inhibited virulence of pathogenic enterococcus species, *E. faecium* and *E. faecalis* with MIC values of >10 to 5 µM, respectively [92]. A similar effect was observed for the *S. aureus* T-SAR12 VRSA strain.

However, the most spectacular effect was observed when the above strains were exposed to a mixture of vancomycin and the lasso peptide (Table 3) [92]. In the case of *E. faecalis*, their exposure to a vancomycin/lasso peptide mixture resulted in an approximately 30-fold decrease in MICs compared to the effect caused by VA alone (Table 3).

The magnitude of the effect was similar for both lasso peptides. The same effect was observed in the case of the VA-resistant *S. aureus* T-SAR12 VRSA.B strain. This demonstrates the synergistic effect of VA and the lasso peptides for *E. faecalis* growth inhibition. In the case of the VA-resistant strains (MICs in the range of hundreds to thousands of µg/mL) of *E. faecium*, resistance-breaking activity was observed after treating them with a VA/Siamycin I mixture (Table 3). The strains became VA-sensitive again with MICs ~ 2 µM. The same effect was observed for the VA-resistant *S. aureus* T-SAR12 VRSA.A strain. Sviceucin showed a significantly weaker or completely absent inhibitory synergistic effect both against VA-resistant *E. faecium* and *S. aureus T-SAR12 VRSA.A* strains. This demonstrates the synergistic effect of VA and the lasso peptide and the ability of both lasso peptides to re-sensitize. In vivo studies have shown that *Galleria mellonella* larvae infected with the *E. faecalis* V583 strain pre-treated with Sviceucin and Siamycin I lasso peptides exhibited significantly lower mortality (to the value of 10–13%) compared to those infected with the strain not pre-treated with lasso peptides (95%) [92]. A similar effect was observed for Sviceucin against other *E. faecalis* strains [92]. This shows the anti-virulence activity of the lasso peptides against *E. faecalis* strains.

## 6. Conclusions

This review highlights the unique structural features, bio- and chemical synthesis approaches and antibiotic properties of lasso peptides. The lasso motif plays a pivotal role in maintaining its structural integrity and biological function. On the one hand, peptides are extremely rewarding structures that are easily amenable to chemical modifications, enabling an extensive exploration of the relationship between their structure and biological activity (SAR). On the other hand, the formation of a macrolactam ring with a C-terminal peptide tail threaded through it is an extremely complex process, the main aspect of which is the enzymatic prefolding of the peptide chain. For this reason, both the synthesis of lasso peptides itself, despite the development of effective heterologous variants of their biosynthesis, and SAR studies are currently facing serious limitations. An example demonstrating the possibility of using chemical synthesis with a cryptand-imidazolium carrier shows that synthetic chemistry is only beginning to show its potential. Looking back at the development of SPPS, there is hope that the non-trivial problems associated with the creation of the lasso system will be solved in the near future. This will open the way both for the synthesis of lasso peptides containing virtually any chemical motif used to modify typical peptides in their structure, as well as for the commercial use of lasso peptides. The discovery of the unusual and unique mechanism of lariocidin’s antibiotic properties, achieved by binding to a site on the ribosome that is atypical for other antibiotics, opens up the potential for this group of lasso peptides to become a new hope for modern medicine in the fight against bacteria, especially those that are multi-drug resistant. In vitro and in vivo studies show that lasso peptides are highly effective against opportunistic strains resistant to methicillin and vancomycin. This bodes well for their potential use as a new weapon against superbugs. Time will tell to what extent these hopes will be fulfilled and how effective this new weapon will prove to be in the millions of-years-long battle against pathogenic bacteria.

## Figures and Tables

**Table 1:** Primary structures and classification of lasso peptides.

Lasso Peptide	Sequence	Class/Ref
Achromosin	GIGSQTWDTIWLWD	II [24]
Acinetodin	GGKGPIFETWVTEGNYYG	II [25]
Actinokineosin	GYPFWDNRDIFGGYTFIG	II [26]
Albusnodin	GQGGGQSEDKRRAYNC	II [27]
Anantin	GFIGWGNDIFGHYSGDF	II [28]
Arcumycin	SGQGWDWVDYHHGWYGWWDD	II [29]
Astexin-1	GLSQGVEPDIGOTYFEESRINQD	II [30]
Astexin-2	GLTQIQALDSVSGQFRDQLGLSAD	II [31]
Astexin-3	GPTPMVGLDSVSGQYWDQHAPLAD	II [31]
Benenodin-1	GVGFGRPDSILTQEQAKPM	II [32]
BI-32169	GLPWGC(&)PSDIPGWNTPWAC(&)	III [33]
Brevunsin	GDMGEEVIEGLVRDSLYPPAG	II [34]
Burhizin	GGAGQYKEVEAGRWSDRIDSDDE	II [35]
Capistruin	GTPGFQTPDARVISRFGFN	II [36]
Cattlecin	SYHWGDYHDWHHGWYGWWDD	II [37]
Caulonodin I	GDVLNAPEPGIGREPTGLSRD	II [35]
Caulonodin II	GDVLFAPEPGVGRPPMGLSED	II [35]
Caulonodin III	GQIYDHPEVGIGAYGCEGLQR	II [35]
Caulonodin IV	SFDVGTIKEGLVSQYYFA	II [35]
Caulonodin V	SIGDSGLRESMSSQTYWP	II [35]
Caulonodin VI	AGTGVLLPETNQIKRYDPA	II [35]
Caulonodin VII	SGIGDVFPEPNMVRRWD	II [35]
Caulosegnin I	GAFVGQPEAVNPLGREIQG	II [38]
Caulosegnin II	GTLTPGLPEDFLPGHYMPG	II [38]
Caulosegnin III	GALVGLLLEDITVARYDPM	II [38]
Chaxapeptin	GFGSKPLDSFGLNFF	II [39]
Citrocin	GGVGKI LEYFIGGGVGRYG	II [40]
Citrulassin	LLGLAGNDRLVLSKN	II [41]
Felipeptin A1	GSRGWGFEPGVRC(&)LIWC(&)D	IV [42]
Felipeptin A2	GGGGRGYEYNKQC(&)LIFC(&)	IV [42]
Fuscanodin	WYTAEWGLELIFVFPRFI	II [43]
Huascopentin	GYGNAWDSKNGLF	II [44]
Humidimycin	C(&1)LGIGSC(&2)DDFAGC(&1)GYAIVC(&2)FW	I [45]
Klebsidin	GSDGPIIEFFNPNGVMHYG	II [46]
Lagmysin	LAGQGSPDLLGGHSLL	II [47]
Lariatin A	GSQLVYREWVGHSNVIKP	II [48]
Lariatin B	GSQLVYREWVGHSNVIKPGP	II [48]
Lariocidin/LAR	SKKSKPGDGKFGRGVKRG	II [49]
Lariocidin B/LAR-B	SK(&) KSKPGDGKFGRGVKR(&)	V* [49]
Lassomycin	GLRRLFADQLVGRRNI	II [50]
Lp2006	GRPNWGFENDWSC(&)VRVC(&)	IV [51]
Microcin J25/MccJ25	GGAGHVPEYFVGIGTPISFYG	II [22]
Moomysin	SYHWGDYHDWHHGWYGWWDD	II [51]
MS-271	C(&1)LGVGSC(&2)NDFAGC(&1)GYAIVC(&2)FW	I [52]
Paeninodin	AGPGTSTPDAFQPDPDEDVHYDS	II [53]
Pandonodin	GVLGNDAEGITLLPLC(&)FKPIC(&)IPTLPPLTGGHA	IV [54]
Propeptin	GYPWWDYRDLFGGHTFISP	II [55]
Pseudomycoidin	AGPGKRLVDQVFEDEDEQGALHHS	II [56]
RES-701-1	GNWHGTAPDWFFNYYW	II [57]
RP 71955/aborycin	C(&1)LGIGSC(&2)NDFAGC(&1)GYAVVC(&2)FW	I [58]
Siamycin I	C(&1)LGVGSC(&2)NDFAGC(&1)GYAIVC(&2)FW	I [59]
Siamycin II	C(&1)LGIGSC(&2)NDFAGC(&1)GYAIVC(&2)FW	I [59]
Specialicin	C(&1)LGVGSC(&2)VDFAGC(&1)GYAVVC(&2)FW	I [60]
Sphingonodin I	GPGGITGDVGLGENNFG	II [35]
Sphingonodin II	GMGSGSTDQNGQPKNLIGG	II [35]
SRO15-2005	GYFVGSYKEYWSRRII	II [61]
SSV-2083	C(&1)VWGGDC(&2)TDFLGC(&1)GTAWIC(&2)V	I [62]
Stlassin	LVVIVQADWNAPGWF	I [63]
Streptomonomycin	SLGSSPYNDILGYPALIVIYP	II [64]
Sungsanpin	GFGSKPIDSFGLSWL	II [65]
Ubonodin	GGDGSIAEYFNRPMHIHDWQIMDSGYYG	II [66]
Ulleungdin	GFIGWGKDIFGHYGG	II [67]

The amino acid residues that form part of the macrolactam ring are marked in green. The ring closes by forming an isopeptide bond between the first (N-terminal) and last amino acid residue (D or E) in this region. & denotes disulfide bridges between C residues. V*—due to the presence of an additional isopeptide bond between the ε-amino group of K2 residue and the carboxyl group of R17 residue, introducing an additional macrocycle (and forming a double-lariat structure) and absent in previously known lasso peptides, the authors of publication [49] propose classifying lariocydin B into a new class (class V) of lasso peptides. In this case & denotes K and R residues participating in the formation of a new isopeptide bond.

**Table 2:** Antibacterial activity of the Lariocidin (LAR) lasso peptide against clinically important bacterial strains [49].

Strain	MIC MHB [µg/mL]	MIC RPMI-1640 [µg/mL]	Notes
*S. aureus* USA300	64	0.5	Superbug (MRSA)
*E. coli* BW25113 *mcr-1*	64	ND	colistin-resistant
*E. coli* C1508	32	1	STEC, MDR
*K. pneumoniae* C1559	>64	4	MDR
*A. baumannii* ATCC17978	128	1	
*A. baumannii* C0286*	8	0.5	Superbag, CRAB

*A. baumannii* C0286*—this strain was used in in vivo studies of the antibacterial activity of LAR; CRAB—carbapenem-resistant *Acinetobacter baumannii*; MHB—cation-adjusted Mueller–Hinton broth; MDR—multi-drug-resistant; MIC—minimum inhibitory concentration; MRSA—methicillin-resistant *S. aureus*; ND—not determined; RPMI-1640—nutrient-limited medium; STEC—Shiga toxin-producing *E. coli*.

**Table 3:** Sviceucin and Siamycin I reverse vancomycin (VA) resistance [92].

Vancomycin (VA) (µg/mL)			
Strain	Control (VA Only)	+Sviceucin (10 µM)	+Siamycin I (2 µM)
*E. faecalis* V583*	32	2	2
*E. faecalis* JH2-2 08048	64	2	<0.5
*E. faecalis* merz96	32	1	2
*S. aureus T-SAR12* VRSA.B	64	2	2
*E. faecium* 1 231 410	128	128	2
*E. faecalis* HIP11704	1024	512	<0.5
*E. faecium* 1 231 502	128	128	2
*E. faecium* 1 230 933	512	256	2
*S. aureus T-SAR12* VRSA.A	>512	>512	2

*E. faecalis* V583* this strain has been used in in vivo studies [92].

## Data Availability

Not applicable.

## Abbreviations

The following abbreviations are used in this manuscript:

AA	amino acid residue
CRAB	carbapenem-resistant *Acinetobacter baumannii*
DMF	N,N-dimethylformamide
LAR-B	Lariocidin B
MccJ25	Microcin J25
MDR	Multi-drug-resistant
MHB	cation-adjusted Mueller–Hinton broth
MIC	minimum inhibitory concentration
MRSA	methicillin-resistant *Staphylococcus aureus*
NMP	N-methylpyrrolidone
PTM	post-translational modification
RiPP	post-translationally modified peptide
RPMI-1640	nutrient-limited medium
RRE	RiPP precursor recognition element
SPPS	solid-phase peptide synthesis
SPS	solid-phase synthesis
STEC	Shiga toxin-producing *E. coli*
WHO BPPL	WHO Bacterial Priority Pathogens List
VA	vancomycin
VRE	vancomycin-resistant enterococci
VRSA	vancomycin-resistant *Staphylococcus aureus*
